# OP58 Diagnostics And Treatments For COVID-19: Update From A Living Systematic Review Of Economic Evaluations

**DOI:** 10.1017/S026646232400120X

**Published:** 2025-01-07

**Authors:** Gareth Hopkin, Jamie Elvidge, Nithin Narayanan, David Nicholls, Dalia Dawoud

## Abstract

**Introduction:**

The COVID-19 pandemic put substantial strain on healthcare systems globally. Early decision-making about diagnostic tests and treatments was driven by the need for rapid responses with a focus on reducing clinical burden. As COVID-19 continues its transition into an endemic state, health technology assessment (HTA) agencies will need to consider the clinical- and cost-effectiveness of tests and treatments, as with other conditions.

**Methods:**

We first conducted a systematic literature review in July 2021 and updated the search in July 2023. The review aimed to identify economic evaluations of diagnostics for SARS-CoV-2 and treatments for COVID-19 using predefined search strategy across journal databases and sources of grey literature. In the update, an additional targeted search was completed with terms relating to novel treatments. Search results were screened by title and abstract, and full texts of potentially relevant studies were reviewed against selection criteria. Studies with very serious methodological limitations were excluded. Findings from studies were synthesized narratively due to high levels of heterogeneity.

**Results:**

The database search identified 8,287 unique records, of which 54 full texts were reviewed, 28 were quality assessed, and 15 were included. Three further studies were included through HTA sources and citation checking. Of the 18 studies ultimately included, 16 evaluated pharmacological treatments including corticosteroids, antivirals, and immunotherapies. Two studies in lower-income settings evaluated the cost-effectiveness of rapid antigen tests and critical care provision. In most studies, a healthcare or payer perspective was used, and the comparator was standard care. There were 17 modeling analyses and one trial-based evaluation. Cost–utility analyses using QALYs were the most common analysis type.

**Conclusions:**

This update indicates that there are cost-effective treatments for COVID-19, with repurposed pharmacological treatments like dexamethasone presenting best value. There also appear to be promising options for people with severe disease alongside standard care. Future economic evaluations would benefit from reflecting the changing context around COVID-19 with parameters that reflect current circumstances, and fully incremental analyses comparing different treatment options.

